# Combination of gold catalysis and Selectfluor for the synthesis of fluorinated nitrogen heterocycles

**DOI:** 10.3762/bjoc.7.162

**Published:** 2011-10-07

**Authors:** Antoine Simonneau, Pierre Garcia, Jean-Philippe Goddard, Virginie Mouriès-Mansuy, Max Malacria, Louis Fensterbank

**Affiliations:** 1 UPMC Univ Paris 06, Sorbonne Universités, Institut Parisien de Chimie Moléculaire (UMR CNRS 7201), 4 place Jussieu, C. 229, 75005 Paris, France

**Keywords:** cycloisomerization reactions, fluorinated pyrrolidines, gold catalysis, Selectfluor

## Abstract

We herein report the synthesis of 3-fluoro-2-methylene-pyrrolidine (**3a**) and -piperidine (**3b**) from 1,5- and 1,6-aminoalkynes, respectively, using a combination of a gold-catalyzed hydroamination reaction followed by electrophilic trapping of an intermediate cyclic enamine by Selectfluor. Careful attention was paid to the elucidation of the mechanism and Selectfluor was suggested to play the double role of promoting the oxidation of gold(I) to a gold(III) active species and also the electrophilic fluorination of the enamine intermediates.

## Introduction

**Scheme 1:**
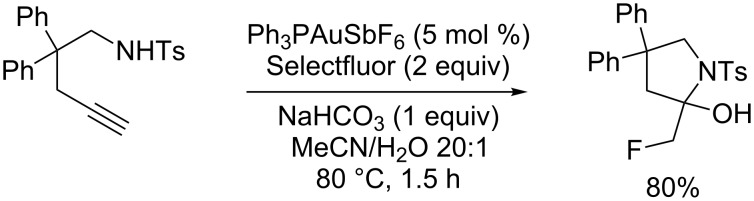
Amino-hydroxyfluorination of alkynes reported by Nevado et al. [2].

The useful properties of fluorinated compounds in medicinal chemistry have motivated an intense effort towards the synthesis of new molecules bearing fluorine substituents [1–2]. Therefore, the development of a rapid access to C–F bonds is of great importance. Quite recently, in their study on the synthesis α-fluoro ketones, Nevado et al. observed the formation of fluorinated pyrrolidinol obtained by a gold-catalyzed cyclization of a 1,5-aminoalkyne in the presence of Selectfluor (Scheme 1) [3]. These authors proposed that the formation of the C(sp^3^)–F bond could be explained either by direct fluorination of the enamine resulting from the gold-promoted alkyne hydroamination or by oxidation of the intermediate vinyl gold(I) complex by Selectfluor into a gold(III) fluoride species followed by a reductive elimination.

Moreover, the formation of C(sp^2^)–F bonds, either by hydrofluorination of alkynes catalyzed by N-heterocyclic carbene gold(I) complexes [4], or by fluorodeauration of transient vinyl gold species [5–6], has been previously reported in the literature.

**Scheme 2:**
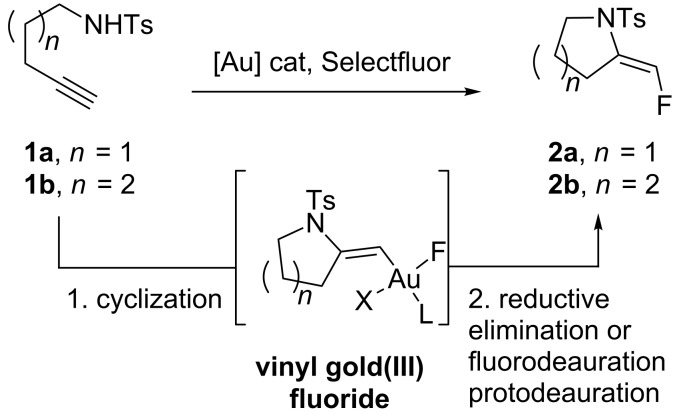
Proposed access to fluoromethylene pyrrolidines and piperidines.

On the basis of our recent results on the gold-catalyzed cyclization of enynes [7–10] and allenylhydrazones [11], as well as the studies from Nevado [3], Hammond and Xu [12], Liu and Xu [13], and Liu [14], we were attracted by the possibility to access fluorinated nitrogen heterocycles **2a** and **2b** by performing subsequently an intramolecular nucleophilic attack of nitrogen onto the gold-activated triple bond on compounds **1a**,**b** in a 5- and 6-exo-dig manner, respectively, and reductive elimination occurring at a vinyl gold(III) fluoride species or bimolecular fluorodeauration (Scheme 2). We also anticipated from this study to gain more insight into the reactivity of gold catalysts/Selectfluor combinations [15–17].

Indeed, pyrrolidine and piperidine skeletons are very attractive ring systems because of their occurrence in numerous biologically active substances, and the design of methodologies allowing the easy introduction of a fluorine atom onto these skeletons could be attractive for medicinal chemists. Recently also, the literature has featured valuable access routes to pyrrolidine promoted by catalytic systems [18–26].

## Results and Discussion

Studies on the scope and limitation of the cyclization–fluorination sequence were carried out with Selectfluor as a source of electrophilic fluorine. Readily available 4-methyl-*N*-(pent-4-ynyl)benzenesulfonamide (**1a**) was used as a model substrate, and all the reactions were performed in anhydrous acetonitrile as the solvent. Various gold catalysts were screened. The results of the optimization of the cyclization reaction conditions are summarized in Table 1.

**Table 1:** Gold catalyst influence on the cyclization of **1a**.^a^

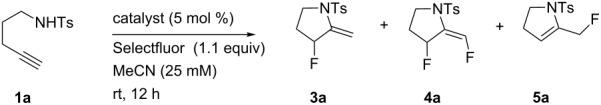				

Entry	Catalyst	**3a** (yield %)	**4a** (yield %)	**5a** (yield %)

1^b^	Ph_3_PAuCl	75	17	0
2^c^	AuCl	46	0	7
3^b^	AuCl_3_	14	2	2
4	IPrAuCl	65	13	0
5^d^	(biphenyl)(*t*-Bu)_2_PAuCl	0	0	0
6	(PhO)_3_PAuCl	35	7	0
7	(2,4-di-*t*-BuPhO)_3_PAuCl	35	5	0
8	(*t*-Bu)_3_PAuCl	35	6	0
9	dppm(AuCl)_2_	43	0	14
10	Ph_3_PAuNTf_2_	35	0	11
11	[Ph_3_PAu]SbF_6_	-	-	-

^a^Products **3a**, **4a** and **5a** were characterized by ^1^H, ^13^C and ^19^F NMR. Compound **4a** was obtained as a single (*E*)-isomer and the configuration of the double bond was confirmed by NOE measurements. ^b^When the reaction was performed without Selectfluor, the starting material was recovered unchanged. ^c^Reaction time: 5 h.^d^An inextricable mixture was obtained.

The reaction of **1a** in the presence of commercially available Ph_3_PAuCl (5 mol %) and Selectfluor (1.1 equiv) in acetonitrile at rt afforded an inseparable mixture of pyrrolidines **3a** in 75% yield and **4a** in 17% yield (Table 1, entry 1). Difluoro derivative **4a** was isolated as a single diastereomer, and its relative stereochemistry was determined by F–F NOE measurement. The expected fluoromethylene tosylpyrrolidine **2a** was not detected. When a AuCl complex was used, product **3a** was obtained in 46% yield with a new monofluorinated 2-pyrroline **5a** in 7% yield (Table 1, entry 2). In the presence of AuCl_3_, a lower yield of **3a** was observed (14%) with trace amounts of **4a** and **5a** (2% yield each, Table 1, entry 3). The use of the *N*,*N*-bis(2,6-diisopropylphenyl)imidazol-2-ylidene (IPr) gold(I) chloride as catalyst led to the formation of **3a** in 65% yield, together with 13% yield of **4a**. Under these conditions, the formation of **5a** was not detected (Table 1, entry 4). Gold(I) phosphite catalysts gave **3a** and **4a** in low yields (Table 1, entries 6 and 7). A similar result was observed with tri(*tert*-butyl)phosphine gold(I) chloride (Table 1, entry 8). The dinuclear complex, dppm(AuCl)_2_, led to **3a** and **5a** in a manner comparable to AuCl (Table 1, entry 9). When we used cationic gold(I) catalyst, Ph_3_PAuNTf_2_, **3a** was obtained in 35% yield with 11% of **5a** (Table 1, entry 10). Finally in the presence of [Ph_3_PAu]SbF_6_ a complex mixture of compounds was obtained (Table 1, entry 11). To the best of our knowledge, these fluorinated pyrrolidines **3a**, **4a** and 2-pyrroline **5a** are unknown and could be interesting building blocks for organic synthesis.

Following the previous catalyst screening, we stuck with the use of PPh_3_AuCl as the catalyst and next investigated the effect of the concentration of **1a** and the stoichiometry of Selectfluor (Table 2).

**Table 2:** Effects of reaction conditions on the Au(I)-catalyzed cyclization of **1a** in the presence of Selectfluor.

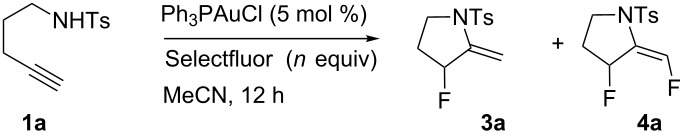					

Entry	*n* (equiv)	Substrate concentration (mM)	Temperature	**3a** (yield %)	**4a** (yield %)

1^a^	1.5	75	reflux	25	25
2	1.5	75	rt	47	17
3	1.1	75	rt	54	13
4^b^	1.1	75	rt	0	0
5	1.1	75	5 °C	17	2
6	1.1	25	rt	75	17
7	1.1	15	rt	73	0

^a^Reaction time: 1 h. ^b^2 equiv of K_2_CO_3_ were added.

Reaction of a 75 mM solution of **1a** with Ph_3_PAuCl (5 mol %) and Selectfluor (1.5 equiv) in acetonitrile under reflux (Table 2, entry 1) afforded an inseparable mixture of **3a** and **4a** both in 25% yield. When the reaction was performed at room temperature, **3a** could be isolated in 47% yield with 17% of **4a** (Table 2, entry 2). Using lower amounts of Selectfluor raised the yield of **3a** to 54% and **4a** to 13% (Table 2, entry 3). In the presence of two equivalents of potassium carbonate the cyclization reaction did not occur (Table 2, entry 4). Lowering the temperature to 5 °C led to a dramatic decrease of the yield of **3a** and **4a** (Table 2, entry 5). Interestingly, the yield of **3a** increased up to 75% when a lower substrate concentration was used (25 mM, Table 2, entry 6). Going to an even more dilute medium resulted in the sole formation of **3a** (Table 2, entry 7). It is noteworthy that in the absence of either the gold catalyst or Selectfluor, the starting material was recovered.

**Scheme 3:**
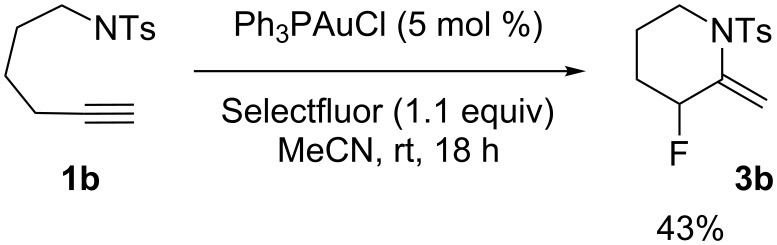
Cyclization of **1b** under standard conditions.

The homologue of **1a**, compound **1b**, was treated with Ph_3_PAuCl (5 mol %) and Selectfluor (1.1 equiv) in acetonitrile at rt. As expected, the 6-exo-dig cyclization occurred and only led to one compound, **3b**, which was isolated in 43% yield (Scheme 3).

**Scheme 4:**
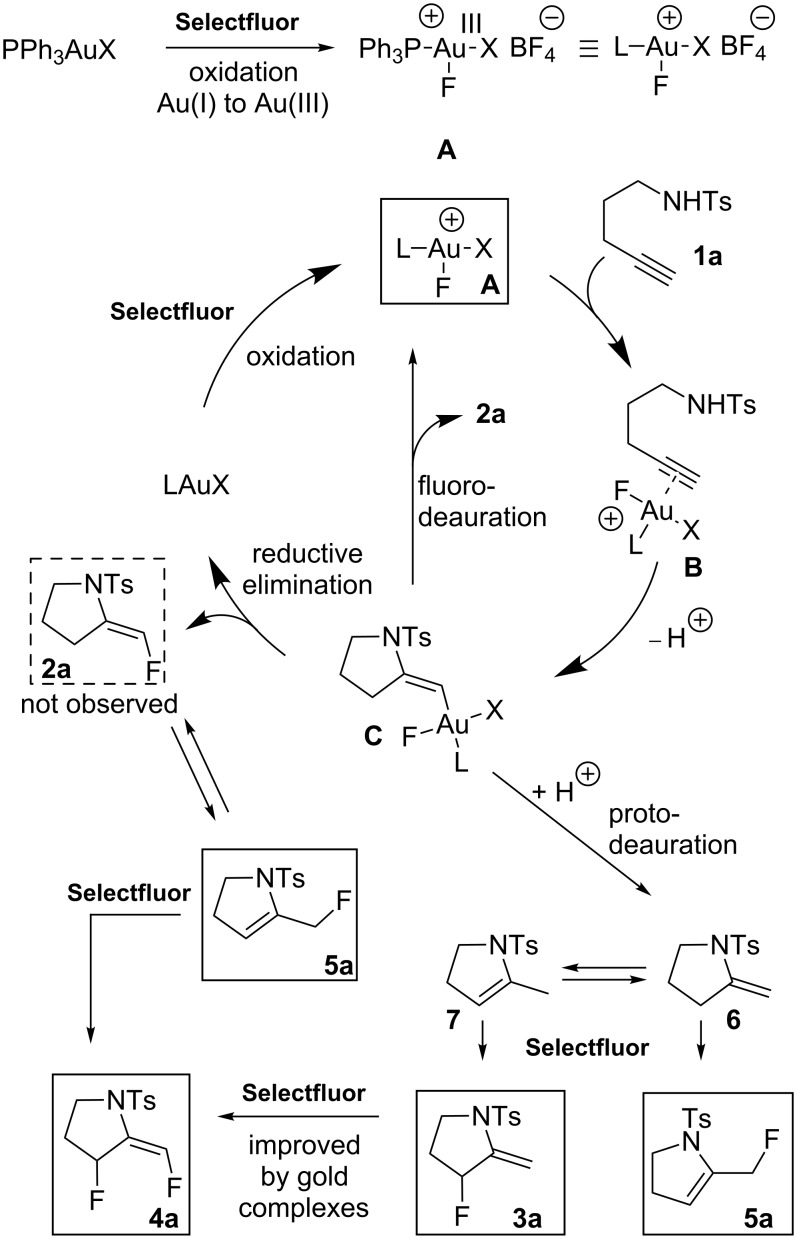
Proposed mechanism.

Our mechanistic proposal for the formation of fluorinated pyrrolidines is outlined in Scheme 4. Oxidation of the Au(I) complex by Selectfluor should give the active cationic Au(III) species **A**. Formation of **A** is consistent with ^19^F NMR experiments analogous to those previously described in the literature [12,27]. Thus, upon addition of Selectfluor to PPh_3_AuCl, a new peak at −181.6 ppm in CD_3_CN was observed that is characteristic of Au(III) species **A** [28]. Coordination of **1a** to **A** would lead to complex **B** in which the coordinated triple bond is activated towards a nucleophilic attack by the NH moiety. The resulting σ-vinyl Au(III) intermediate **C** could undergo a reductive elimination of its σ-vinyl and F ligands to give **2a**, or a protodeauration leading to pyrrolidine **6**, which would also rapidly isomerize into **7**. Both **6** and **7** under the given reaction conditions would evolve to **3a** and **5a**.

**Scheme 5:**
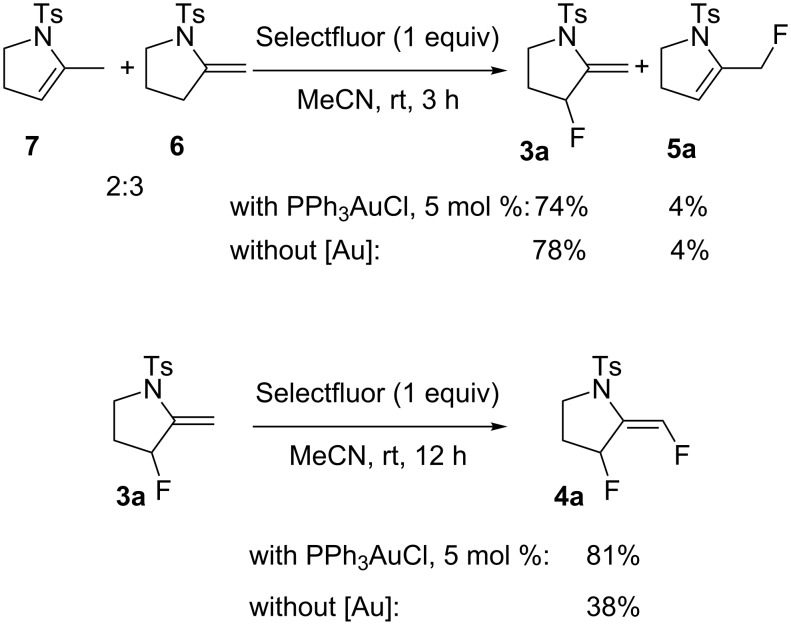
Mechanistic probes.

The following experiments were performed to probe the mechanism proposed in Scheme 4. The reaction of a mixture of **6** and **7**, in a 2:3 ratio, with PPh_3_AuCl (5 mol %) and Selectfluor (1 equiv) in acetonitrile at rt during 3 h, led to a mixture of **3a** and **5a** with yields of 74% and 4%, respectively. In the absence of a gold catalyst, a similar result was obtained (Scheme 5) confirming that Selectfluor itself can react with **6** and **7** to give **3a** and **5a**, which is consistent with Shreeve’s study on the fluorination of enamines [29].

However, the formation of **5a** may also be ascribed to the reductive elimination of **C**, which leads to **2a**, and then further isomerization of the double bond of **2a** (Scheme 4). As far as the formation of **4a** is concerned, we found that treatment of **3a** with one equivalent of Selectfluor in the presence of Ph_3_PAuCl (5 mol %), led to **4a** in 81% yield. Nevertheless, in the absence of Au(I) catalyst, **4a** was also formed but with a lower yield of 38% (Scheme 5). These results suggest that the formation of **4a** may not exclusively result from the Selectfluor-mediated fluorination of **3a**. These findings are consistent with Gouverneur's study [5], which showed a competition between fluorodeauration and protodeauration. In our case, protodeauration appears to be the major, if not the exclusive, pathway.

**Scheme 6:**
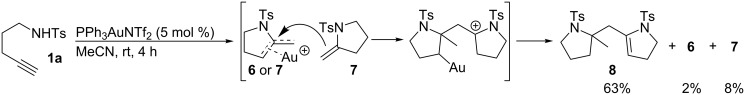
Cationic Au(I)-catalyzed reaction of **1a** without Selectfluor.

In a final experiment, **1a** was treated with PPh_3_AuNTf_2_, without Selectfluor, and dimer **8** was formed in 63% yield along with **6** and **7** in low yields of 2% and 8%, respectively. The formation of **8** could be explained as outlined in Scheme 6. The cationic Au(I) catalyst would promote the cyclization of **1a** to the mixture of enamines **6** and **7** as previously observed with cationic Au(III) species (Scheme 4). Activation of the electron rich double bond of **6** or **7** by the cationic Au(I) complex could finally trigger the dimerization and so the formation of **8**.

## Conclusion

In conclusion, we have reported a gold-catalyzed synthesis of fluorinated pyrrolidines from 1,ω-aminoalkynes using Selectfluor as the source of fluorine. This method allows a rapid, efficient and mild conversion of readily available aminoalkynes into valuable nitrogen heterocycles substituted by a fluorine atom in position 3 of the ring. This could certainly be applied to the synthesis of biologically relevant substrates. Current efforts are being made in this direction and a more exemplified study will be reported in due course.

## Experimental

### 

#### General methods

Acetonitrile was distilled over calcium hydride. Other reagents were commercially available and used without further purification. Thin layer chromatography (TLC) was performed on Merck 60 F_254_ silica gel. Acros aluminium oxide, basic, Brockmann I, 50–200 µm, 60A was used for column chromatography. NMR spectra (^1^H, ^13^C, DEPT, COSY, HMQC, HMBC, NOE) were recorded at room temperature at 300 or 400 MHz on a Bruker AVANCE spectrometer. Chemical shifts are given in ppm, referenced to the residual proton resonance of the solvents (δ = 7.26 for CHCl_3_) or to the residual carbon resonance of the solvent (δ = 77.16 for CDCl_3_). Coupling constants (*J*) are given in Hertz (Hz). The terms m, s, d, t and q refer to multiplet, singlet, doublet, triplet and quartet; br means that the signal is broad. When possible, ^1^H and ^13^C signals were assigned on the basis of DEPT and 2D NMR (COSY, HMBC) experiments. Low-resolution mass spectra (MS) and high-resolution mass spectra (HRMS) were measured on a Bruker MicroTOF mass spectrometer. Infrared spectra (IR) were recorded on a Bruker Tensor 27 spectrometer and melting points were measured on a Wagner & Munz HEIZBANK Kofler bench.

#### General procedure for the synthesis of precursors


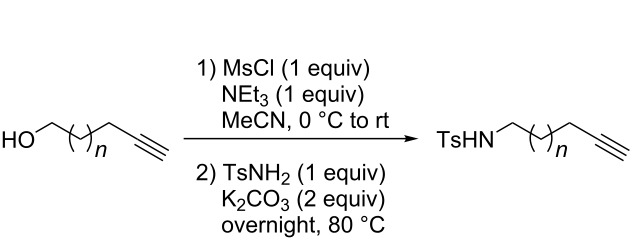


To a cold solution (0 °C) of the starting alcohol in MeCN, MsCl and Et_3_N were successively added, and the mixture was warmed to rt. After 1 h, K_2_CO_3_ and TsNH_2_ were added and the mixture was warmed at 80 °C overnight. Once back to rt, the mixture was directly purified by flash chromatography being eluted first with petroleum ether and then with petroleum ether/ethyl acetate 9:1.

#### Spectral data of cyclization precursors


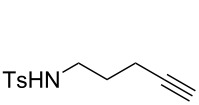


**1a.** In agreement with the literature data [30–31].


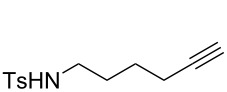


**1b.** In agreement with the literature data [31–32].

#### General procedure for the cyclization reaction


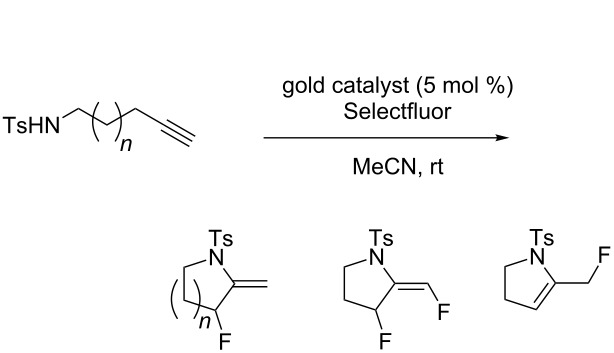


Into an oven-dried Schlenk apparatus, the Selectfluor (0.33 mmol, 117 mg, 1.1 equiv) and the gold catalyst (16 µmol, 0.05 equiv) were loaded under a flow of argon. These solids were dried under vacuum at 70–80 °C for 2 h. The aminoalkyne (0.30 mmol, 1 equiv) was then added, followed by anhydrous MeCN (12 mL), under a flow of argon. The mixture was stirred at rt until complete consumption of the starting material was observed by TLC. The reaction was then filtered on a short plug of basic alumina. After removal of the solvents under reduced pressure, the crude product was purified by flash column chromatography on alumina with pentane/ethyl acetate 85:15 as eluent.

#### Spectral data of cyclic products


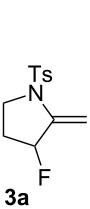


**3a.** White solid, mp 102 °C; ^1^H NMR (400 MHz, CDCl_3_) δ 7.73 (d, *J* = 8.3 Hz, 2H), 7.30 (d, *J* = 8.1 Hz, 2H), 5.38 (d, *J* = 5.2 Hz, 1H), 5.09 (ddd, *J*_H-F_ = 54.6, *J*_H-H_ = 4.2, 1.9 Hz, 1H), 4.76 (d, *J* = 6.0 Hz, 1H), 3.88–3.82 (m, 1H), 3.63 (td, *J* = 9.8, 6.5 Hz, 1H), 2.42 (s, 3H), 2.14–1.87 (m, 2H); ^19^F NMR (376.5 MHz, CDCl_3_) δ −168.4 (m, 1F); ^13^C NMR (101 MHz, CDCl_3_) δ 144.5 (C), 143.2 (d, *J*_C-F_ = 15.4 Hz, C), 134.2 (C), 129.7 (2CH), 127.6 (2CH), 97.2 (d, *J*_C-F_ = 8.4 Hz, CH_2_), 93.7 (d, *J*_C-F_ = 178.5 Hz, CH), 48.6 (CH_2_), 29.5 (d, *J*_C-F_ = 22.3 Hz, CH_2_), 21.7 (CH_3_); IR (neat) 2358, 1652, 1338, 1251, 1156, 1085, 1000, 866, 814, 651 cm^−1^; HRMS (*m*/*z*): [M + Na]^+^ calcd for C_12_H_14_FNO_2_S, 278.0621; found, 278.0632.


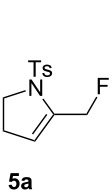


**5a.** Isolated as a minor product in mixture with **3a**; ^1^H NMR (400 MHz, CDCl_3_) δ 7.71 (d, *J* = 8.7 Hz, 2H), 7.32 (d, *J* = 8.7 Hz, 2H), 5.39–5.37 (m, 1H), 5.22 (d, *J*_H-F_ = 46.8 Hz, 2H), 3.78 (t, *J* = 8.9 Hz, 2H), 2.43 (s, 3H), 2.33–2.22 (m, 2H); ^19^F NMR (376 MHz, CDCl_3_) δ −213.6 to −213.9 (m, 1F); ^13^C NMR (101 MHz, CDCl_3_) δ 144.1 (C), 139.5 (d, *J*_C-F_ = 20.7 Hz, C), 134.1 (C), 130.1 (2CH), 127.8 (2CH), 115.4 (d, *J*_C-F_ = 8.1 Hz, CH), 78.5 (d, *J*_C-F_ = 167.4 Hz, CH_2_), 50.4 (CH_2_), 27.7 (CH_2_), 21.7 (CH_3_).


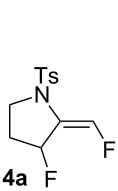


**4a.** White solid, mp 68 °C; ^1^H NMR (400 MHz, CDCl_3_) δ 7.72 (d, *J* = 8.3 Hz, 2H), 7.39 (dd, *J*_H-F_ = 80.7, *J*_H-H_ = 6.1 Hz, 1H), 7.32 (d, *J* = 8.1 Hz, 2H), 5.63 (dt, *J*_H-F_ = 53.8 Hz, *J*_H-H_ = 3.2 Hz, 1H), 3.83 (t, *J* = 9.2 Hz, 1H), 3.48 (ddd, *J* = 11.1, 10.1, 6.2 Hz, 1H), 2.43 (s, 3H), 2.09 (ddd, *J* = 17.5, 14.4, 6.1 Hz, 1H), 1.97–1.76 (m, 1H); ^19^F NMR (376 MHz, CDCl_3_) δ −149.5 (dd, *J*_H-F_ = 80.7, *J*_F-F_ = 8.9 Hz, 1F), −171.6 to −173.7 (m, 1F); ^13^C NMR (101 MHz, CDCl_3_) δ 144.8 (C), 142.0 (dd, *J*_C-F_ = 250.4, 10.1 Hz, CH), 133.3 (C), 129.9 (2CH), 128.2 (C), 127.8 (2CH), 88.1 (dd, *J*_C-F_ = 178.7, 3.5 Hz, CHF), 48.7 (CH_2_), 30.4 (d, *J*_C-F_ = 23.1 Hz, CH_2_), 21.8 (CH_3_); IR (neat) 2359, 1597, 1353, 1163, 1133, 1090, 1060, 1011, 964, 814, 664 cm^−1^; HRMS (*m*/*z*): [M + Na]^+^ calcd for C_12_H_13_F_2_NO_2_S, 296.0527; found, 296.0535.


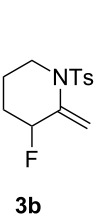


**3b.** Colorless oil; ^1^H NMR (400 MHz, CDCl_3_) δ 7.73 (d, *J* = 8.3 Hz, 2H), 7.28 (d, *J* = 8.0 Hz, 2H), 5.35 (s, 1H), 5.16 (s, 1H), 4.70 (dt, *J*_H-F_ = 49.6 Hz, *J*_H-H_ = 5.3 Hz, 1H), 3.68–3.54 (m, 2H), 2.42 (s, 3H), 1.97–1.81 (m, 2H), 1.73 (m, 1H), 1.61–1.48 (m, 1H); ^19^F NMR (376 MHz, CDCl_3_) δ −173.4 (m, 1F); ^13^C NMR (101 MHz, CDCl_3_) δ 143.7 (C), 140.5 (d, *J*_C-F_ = 19.0 Hz, C), 137.3 (C), 129.7 (2CH), 127.7 (2CH), 110.7 (d, *J*_C-F_ = 7.7 Hz, CH_2_), 88.4 (d, *J*_C-F_ = 178.6 Hz, CHF), 46.9 (CH_2_), 30.7 (d, *J*_C-F_ = 21.7 Hz, CH_2_), 21.7 (CH_3_), 20.9 (d, *J*_C-F_ = 5.4 Hz, CH_2_); IR (neat) 1647, 1598, 1451, 1340, 1157, 1098, 1057, 950, 908, 814, 690, 653 cm^−1^; HRMS (*m*/*z*): [M + Na]^+^ calcd for C_13_H_16_FNO_2_S, 292.0778; found, 292.0776.


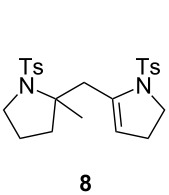


**8.** White solid, mp 66 °C; ^1^H NMR (300 MHz, CDCl_3_) δ 7.74 (d, *J* = 8.2 Hz, 2H), 7.65 (d, *J* = 8.1 Hz, 2H), 7.30 (d, *J* = 5.2 Hz, 2H), 7.28 (d, *J* = 6.3 Hz, 2H), 5.41 (br s, 1H), 3.89–3.69 (m, 2H), 3.54–3.46 (m, 1H), 3.30–3.23 (m, 3H), 2.66–2.52 (m, 1H), 2.44 (s, 3H), 2.42 (s, 3H), 2.01–1.87 (m, 3H), 1.78–1.58 (m, 2H), 1.41 (s, 3H); ^13^C NMR (75 MHz, CDCl_3_) δ 143.9 (C), 142.8 (C), 140.9 (C), 138.8 (C), 134.1 (C), 129.7 (2CH), 129.5 (2CH), 127.8 (2CH), 127.3 (2CH), 119.5 (CH), 68.20 (C), 51.3 (CH_2_), 49.9 (CH_2_), 39.6 (CH_2_), 39.2 (CH_2_), 27.7 (CH_2_), 26.6 (CH_3_), 22.7 (CH_2_), 21.7 (CH_3_), 21.6 (CH_3_); IR (neat) 1452, 1332, 1154, 1089, 1000, 811, 655 cm^−1^; HRMS (*m*/*z*): [M + Na]^+^ calcd for C_24_H_30_N_2_O_4_S_2_, 497.1539; found, 497.1517.

#### Synthesis of cyclic enamines


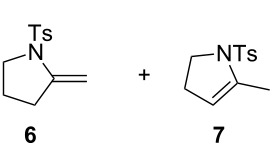


Cyclic enamines were synthesized in 59% yield (endo/exo 2:3) according to a literature procedure [32]. Spectral data matched those reported.

#### General procedure for the fluorination reactions of the enamines


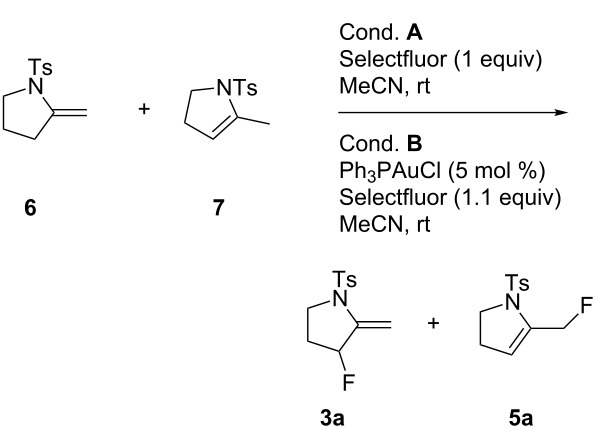


Conditions **A**: In an oven-dried Schlenk apparatus the Selectfluor (0.17 mmol, 1 equiv) was loaded under a flow of argon. This was then dried under vacuum at 70–80 °C for 2 h. The mixture of cyclic enamines (0.17 mmol, 40 mg, 1 equiv) was then added, followed by anhydrous MeCN (7 mL), under a flow of argon. The mixture was stirred at rt until complete consumption of the starting material was observed by TLC. The reaction mixture was then filtered on a short plug of basic alumina. After removal of the solvents under reduced pressure, the crude product was purified by flash column chromatography on alumina with pentane/ethyl acetate 85:15 as eluent.

Conditions **B**: In an oven-dried Schlenk apparatus, the Selectfluor (0.33 mmol, 117 mg, 1.1 equiv) and triphenylphosphine gold chloride (16 µmol, 7.4 mg, 0.05 equiv) were loaded under a flow of argon. These solids were dried under vacuum at 70–80 °C for 2 h. A mixture of cyclic enamines (0.3 mmol, 72 mg, 1 equiv) was then added, followed by anhydrous MeCN (12 mL), under a flow of argon. The mixture was stirred at rt until complete consumption of the starting material was observed by TLC. The reaction was then filtered on a short plug of basic alumina. After removal of the solvents under reduced pressure, the crude product was purified by flash column chromatography on alumina with pentane/ethyl acetate 85:15 as eluent.

## Supporting Information

File 1 ^1^H, ^13^C, ^19^F NMR spectra of products **3a**, **4a**, **5a**, **3b** and **8**.
