# Supplementary material for: Combination of gold catalysis and Selectfluor for the synthesis of fluorinated nitrogen heterocycles
Source: Beilstein J Org Chem. 2011 Oct 7;7:1379–86. doi: 10.3762/bjoc.7.162 (PMC3201052; doi:10.3762/bjoc.7.162)
Supplement: File 1 — 1H, 13C, 19F NMR spectra of products 3a, 4a, 5a, 3b and 8. [file Beilstein_J_Org_Chem-07-1379-s001.pdf]

# Supporting Information

## for

# Combination of gold catalysis and Selectfluor for the synthesis of fluorinated nitrogen heterocycles

Antoine Simonneau, Pierre Garcia, Jean-Philippe Goddard, Virginie Mouriès-Mansuy, Max Malacria* and Louis Fensterbank*

Address: UPMC Univ Paris 06, Sorbonne Universités, Institut Parisien de Chimie Moléculaire (UMR CNRS 7201), 4 place Jussieu, C. 229, 75005 Paris, France.

Email: Max Malacria* - max.malacria@upmc.fr;

Louis Fensterbank* - louis.fensterbank@upmc.fr

* Corresponding author

$^{1}H$, $^{13}C$, $^{19}F$ NMR spectra of products **3a**, **4a**, **5a**, **3b** and **8**.

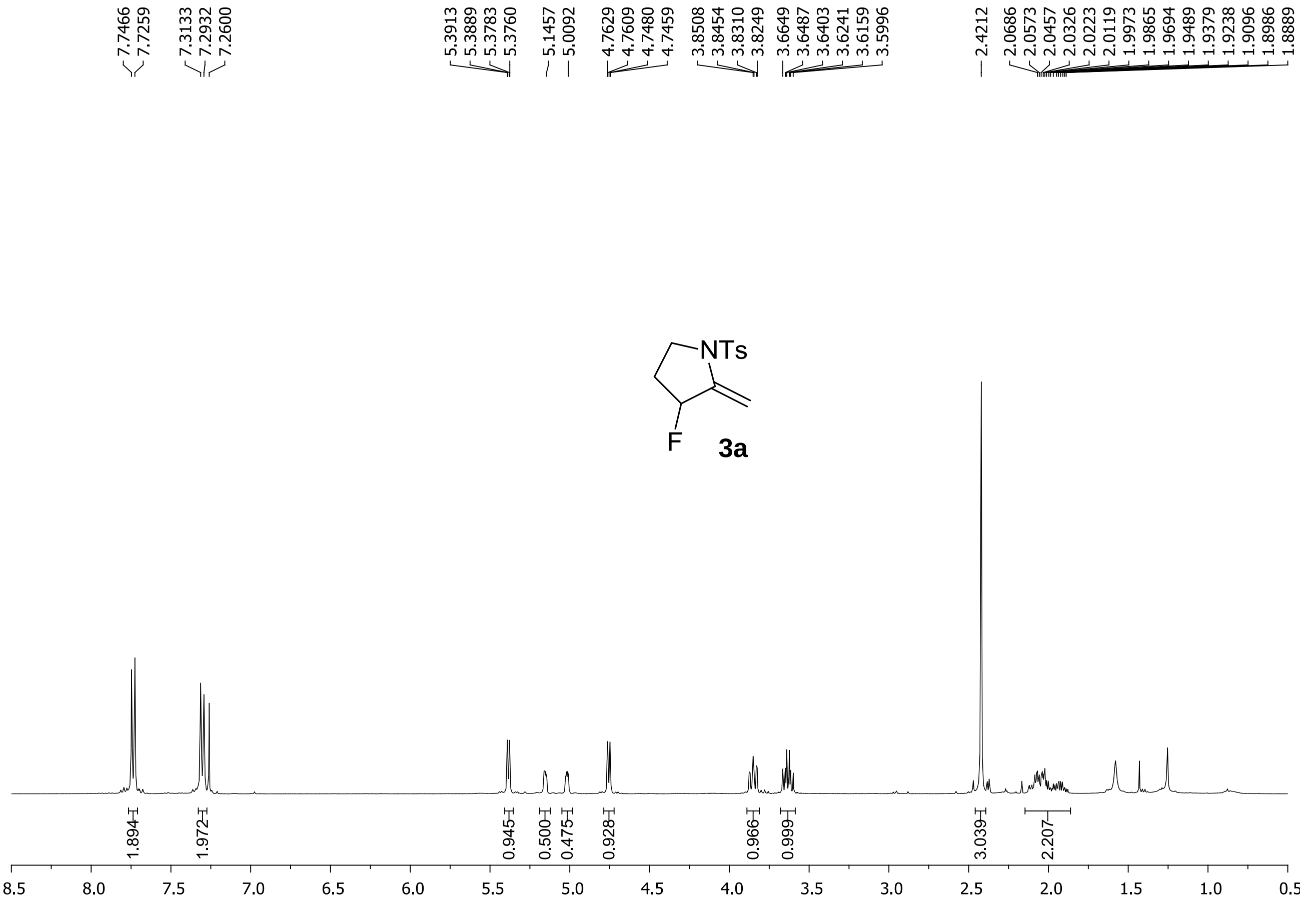


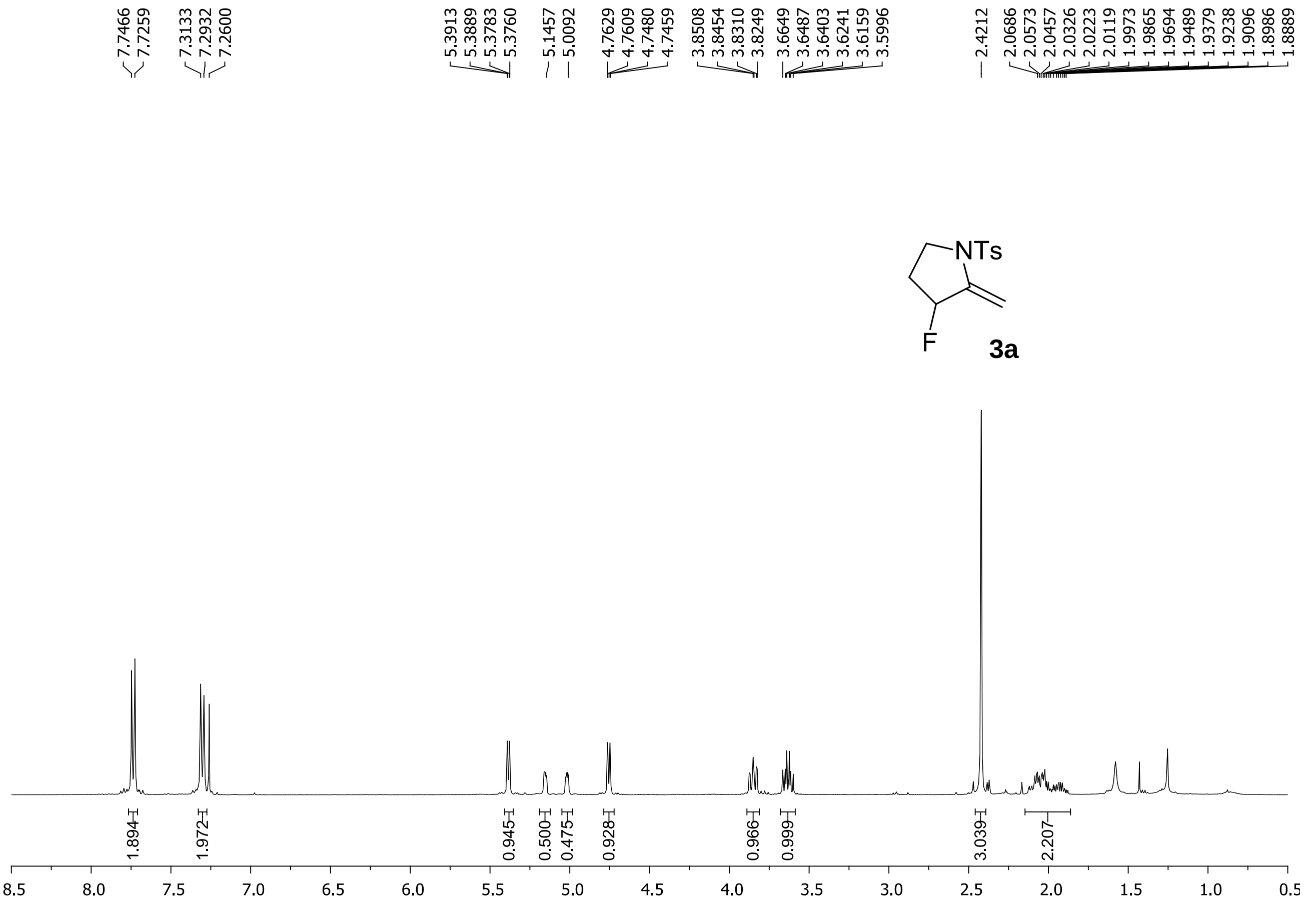
7.7466
7.7259
7.3133
7.2932
7.2600
5.3913
5.3889
5.3783
5.3760
5.1457
5.0092
4.7629
4.7609
4.7480
4.7459
3.8508
3.8454
3.8310
3.8249
3.6649
3.6487
3.6403
3.6241
3.6159
3.5996
2.4212
2.0686
2.0573
2.0457
2.0326
2.0223
2.0119
1.9973
1.9865
1.9694
1.9489
1.9379
1.9238
1.9096
1.8986
1.8889
NTs
F
3a
1.894
1.972
0.945
0.500
0.475
0.928
0.966
0.999
3.039
2.207
8.5 8.0 7.5 7.0 6.5 6.0 5.5 5.0 4.5 4.0 3.5 3.0 2.5 2.0 1.5 1.0 0.5

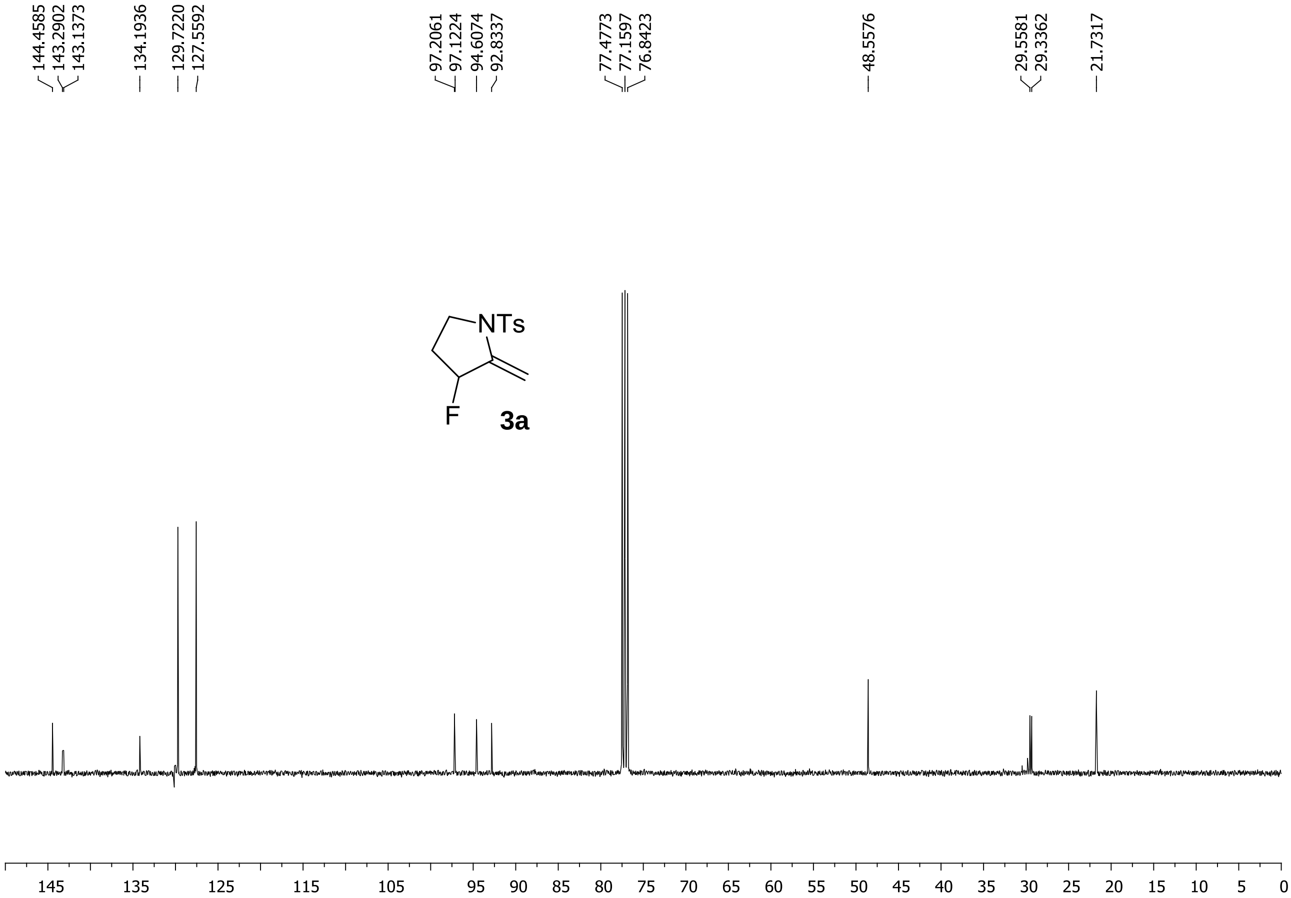
144.4585
143.2902
143.1373
134.1936
129.7220
127.5592
97.2061
97.1224
94.6074
92.8337
77.4773
77.1597
76.8423
48.5576
29.5581
29.3362
21.7317
NTs
F
3a
145 135 125 115 105 95 90 85 80 75 70 65 60 55 50 45 40 35 30 25 20 15 10 5 0

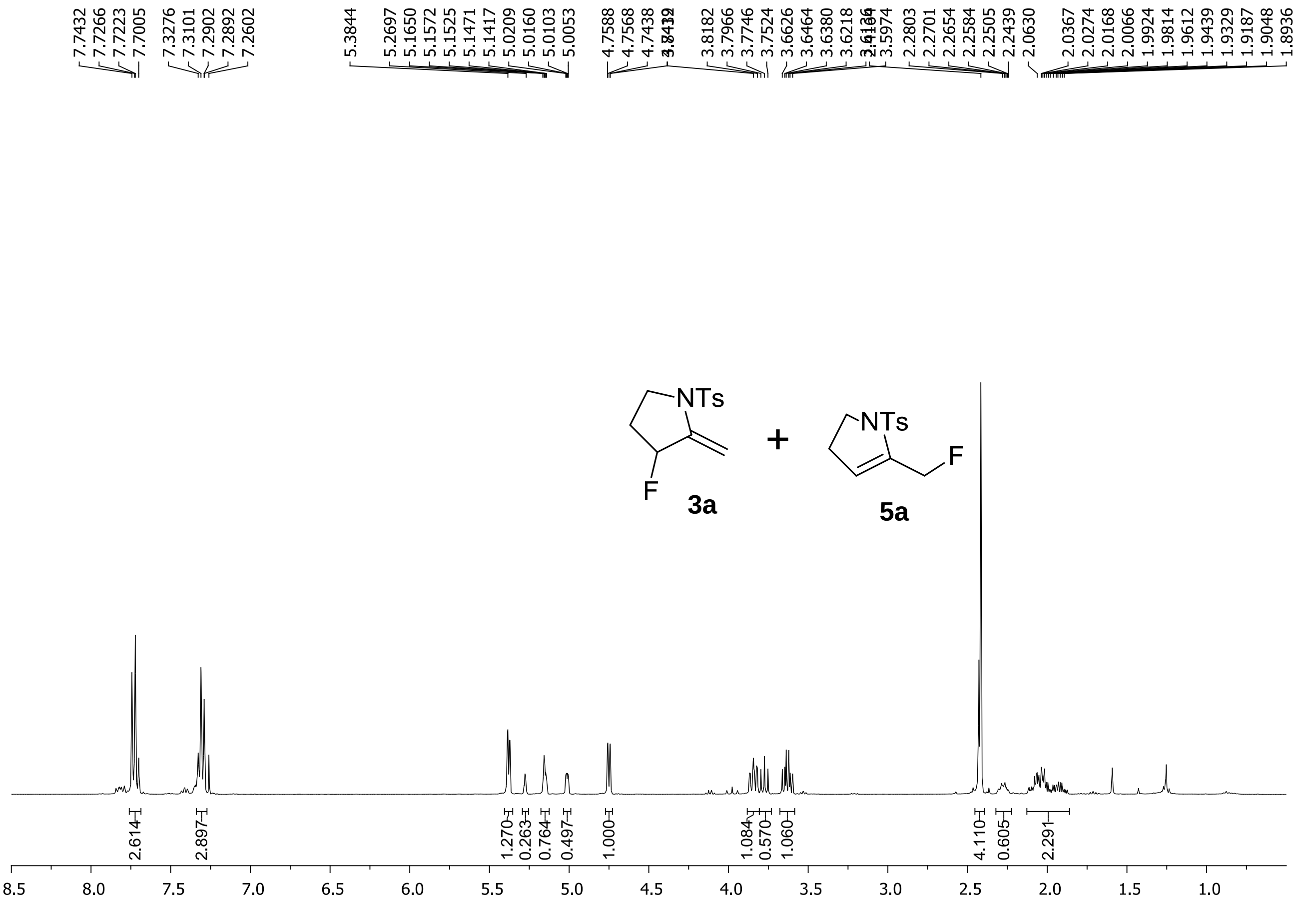
7.7432
7.7266
7.7223
7.7005
7.3276
7.3101
7.2902
7.2892
7.2602
5.3844
5.2697
5.1650
5.1572
5.1525
5.1471
5.1417
5.0209
5.0160
5.0103
5.0053
4.7588
4.7568
4.7438
3.8182
3.7966
3.7746
3.7524
3.6626
3.6464
3.6380
3.6218
3.5974
2.2803
2.2701
2.2654
2.2584
2.2505
2.2439
2.0630
2.0367
2.0274
2.0168
2.0066
1.9924
1.9814
1.9612
1.9439
1.9329
1.9187
1.9048
1.8936
NTs
F
3a
+
NTs
F
5a
2.614
2.897
1.270
0.263
0.764
0.497
1.000
1.084
0.570
1.060
4.110
0.605
2.291
8.5 8.0 7.5 7.0 6.5 6.0 5.5 5.0 4.5 4.0 3.5 3.0 2.5 2.0 1.5 1.0

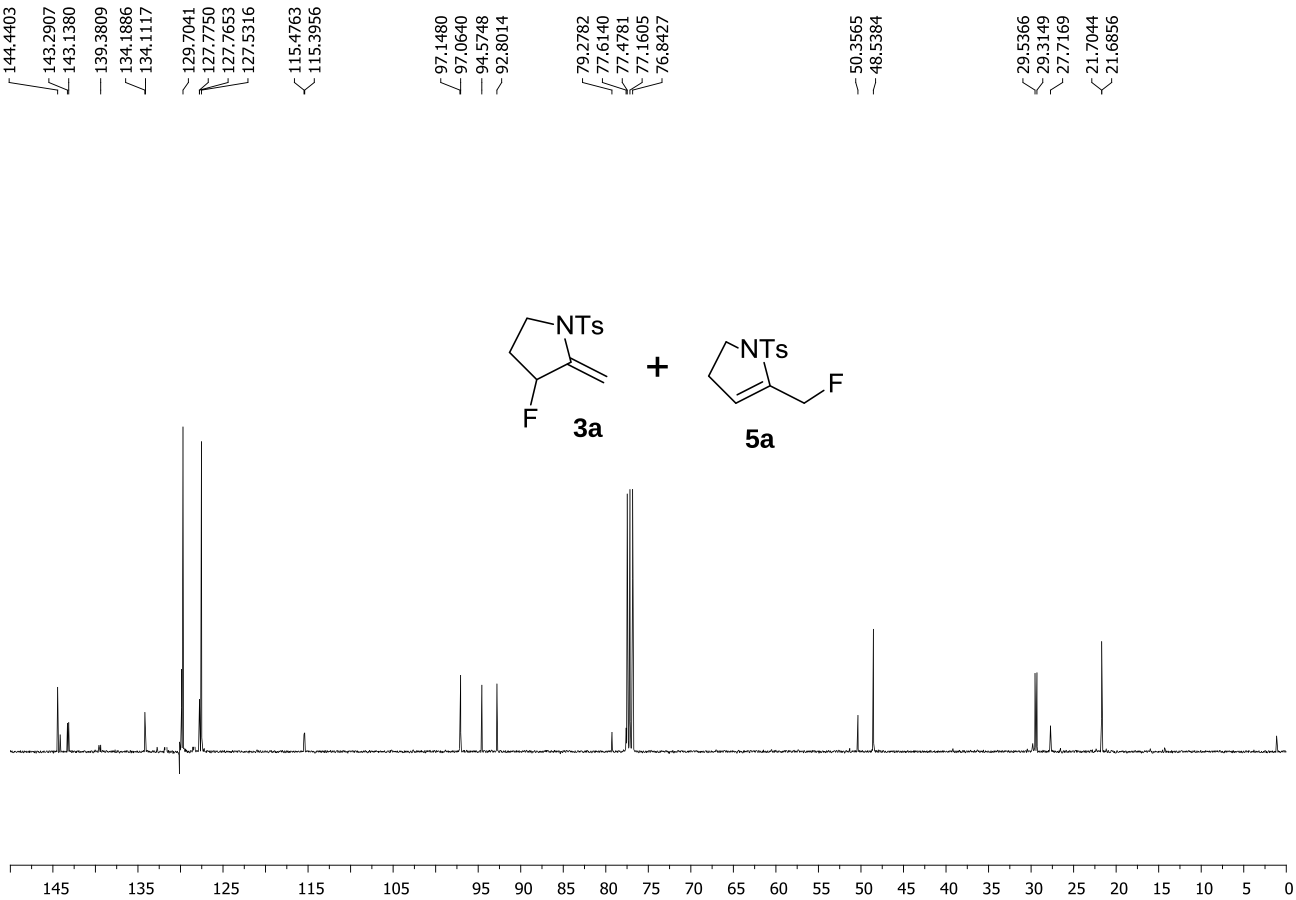
144.4403
143.2907
143.1380
139.3809
134.1886
134.1117
129.7041
127.7750
127.7653
127.5316
115.4763
115.3956
97.1480
97.0640
94.5748
92.8014
79.2782
77.6140
77.4781
77.1605
76.8427
50.3565
48.5384
29.5366
29.3149
27.7169
21.7044
21.6856
NTs
F
3a
+
NTs
F
5a
145
135
125
115
105
95
90
85
80
75
70
65
60
55
50
45
40
35
30
25
20
15
10
5
0

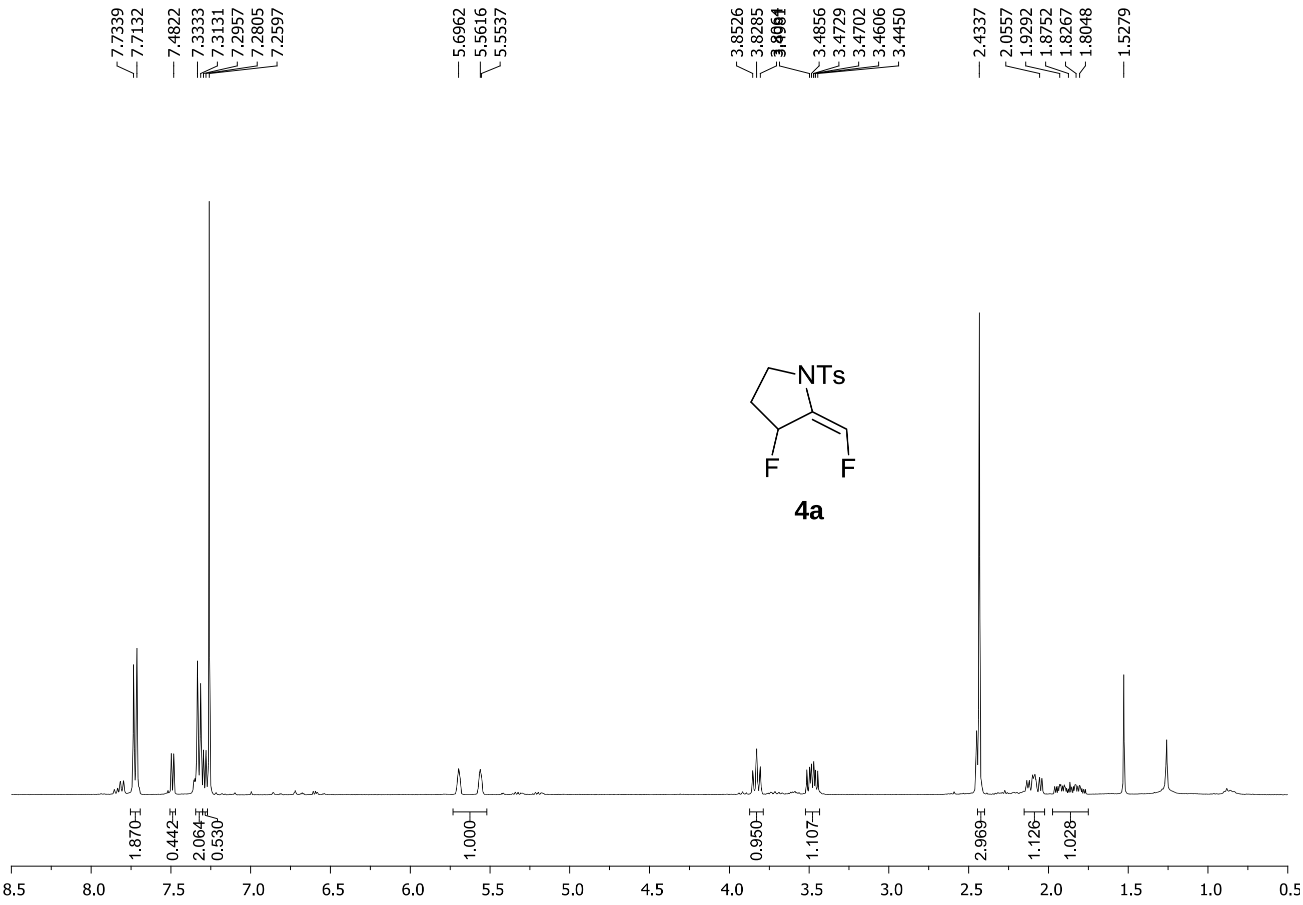
7.7339
7.7132
7.4822
7.3333
7.3131
7.2957
7.2805
7.2597
5.6962
5.5616
5.5537
3.8526
3.8285
3.4856
3.4729
3.4702
3.4606
3.4450
2.4337
2.0557
1.9292
1.8752
1.8267
1.8048
1.5279
NTs
F
F
4a
1.870
0.442
2.064
0.530
1.000
0.950
1.107
2.969
1.126
1.028
8.5
8.0
7.5
7.0
6.5
6.0
5.5
5.0
4.5
4.0
3.5
3.0
2.5
2.0
1.5
1.0
0.5

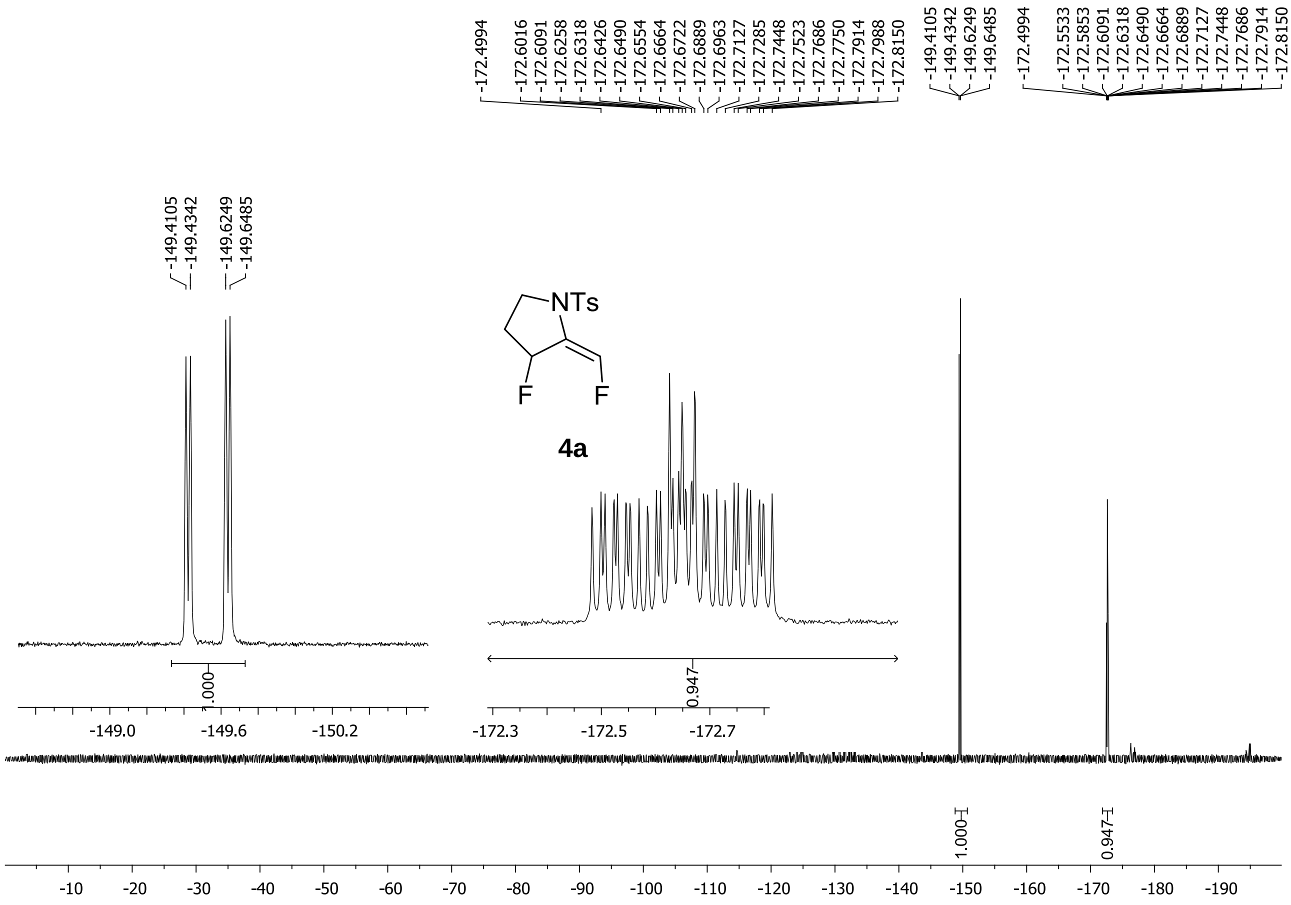
-172.4994
-172.6016
-172.6091
-172.6258
-172.6318
-172.6426
-172.6490
-172.6554
-172.6664
-172.6722
-172.6889
-172.6963
-172.7127
-172.7285
-172.7448
-172.7523
-172.7686
-172.7750
-172.7914
-172.7988
-172.8150
-149.4105
-149.4342
-149.6249
-149.6485
-172.4994
-172.5533
-172.5853
-172.6091
-172.6318
-172.6490
-172.6664
-172.6889
-172.7127
-172.7448
-172.7686
-172.7914
-172.8150
-149.4105
-149.4342
-149.6249
-149.6485
NTs
F
F
4a
1.000
-149.0
-149.6
-150.2
0.947
-172.3
-172.5
-172.7
1.000
0.947
-10 -20 -30 -40 -50 -60 -70 -80 -90 -100 -110 -120 -130 -140 -150 -160 -170 -180 -190

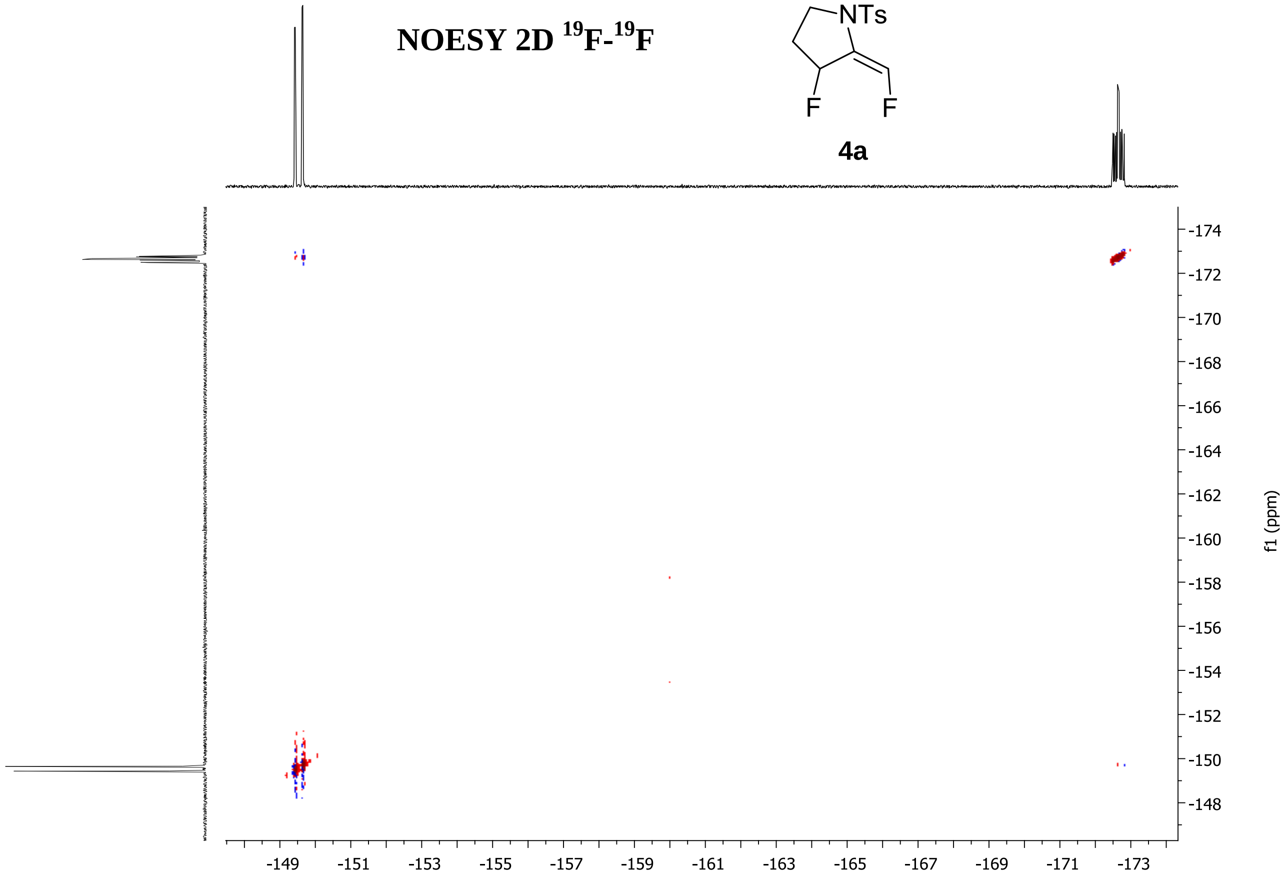
NOESY 2D $^{19}$F-$^{19}$F
NTs
F
F
4a
-174 -172 -170 -168 -166 -164 -162 -160 -158 -156 -154 -152 -150 -148
f1 (ppm)
-149 -151 -153 -155 -157 -159 -161 -163 -165 -167 -169 -171 -173

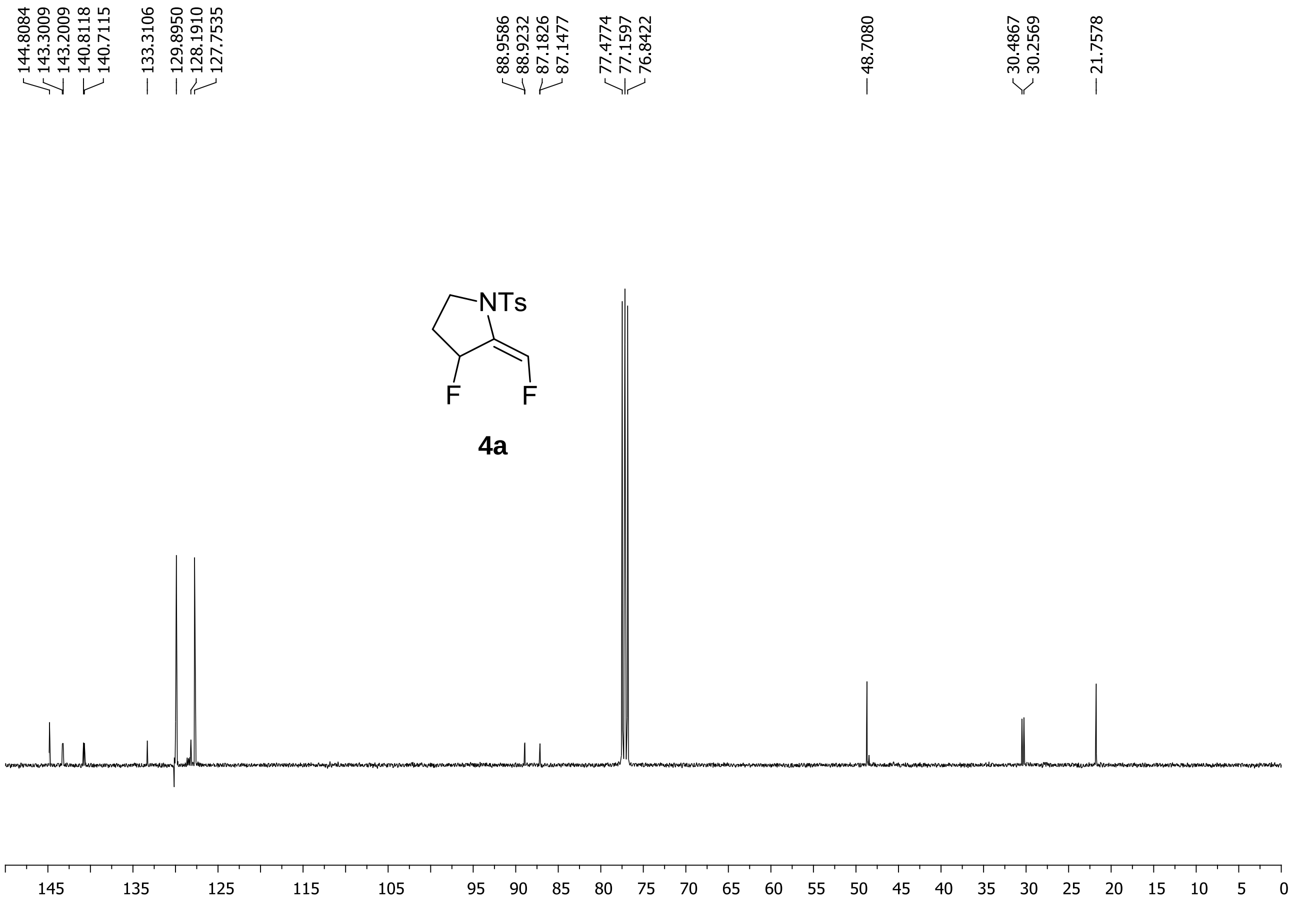
144.8084
143.3009
143.2009
140.8118
140.7115
133.3106
129.8950
128.1910
127.7535
88.9586
88.9232
87.1826
87.1477
77.4774
77.1597
76.8422
48.7080
30.4867
30.2569
21.7578
NTs
F
F
4a
145 135 125 115 105 95 90 85 80 75 70 65 60 55 50 45 40 35 30 25 20 15 10 5 0

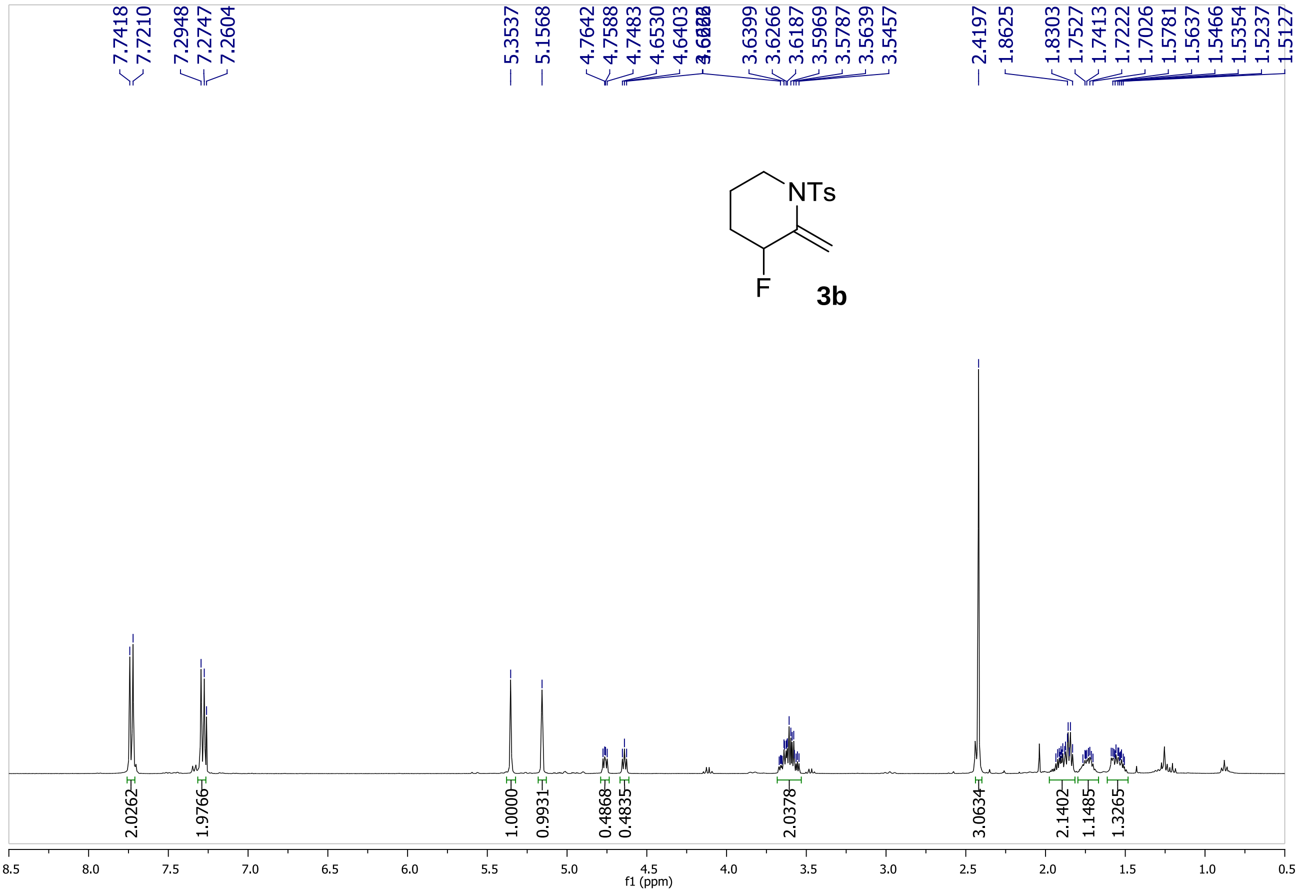
7.7418
7.7210
7.2948
7.2747
7.2604
5.3537
5.1568
4.7642
4.7588
4.7483
4.6530
4.6403
3.6399
3.6266
3.6187
3.5969
3.5787
3.5639
3.5457
2.4197
1.8625
1.8303
1.7527
1.7413
1.7222
1.7026
1.5781
1.5637
1.5466
1.5354
1.5237
1.5127
NTs
F
3b
2.0262
1.9766
1.0000
0.9931
0.4868
0.4835
2.0378
3.0634
2.1402
1.1485
1.3265
8.5 8.0 7.5 7.0 6.5 6.0 5.5 5.0 4.5 4.0 3.5 3.0 2.5 2.0 1.5 1.0 0.5
f1 (ppm)

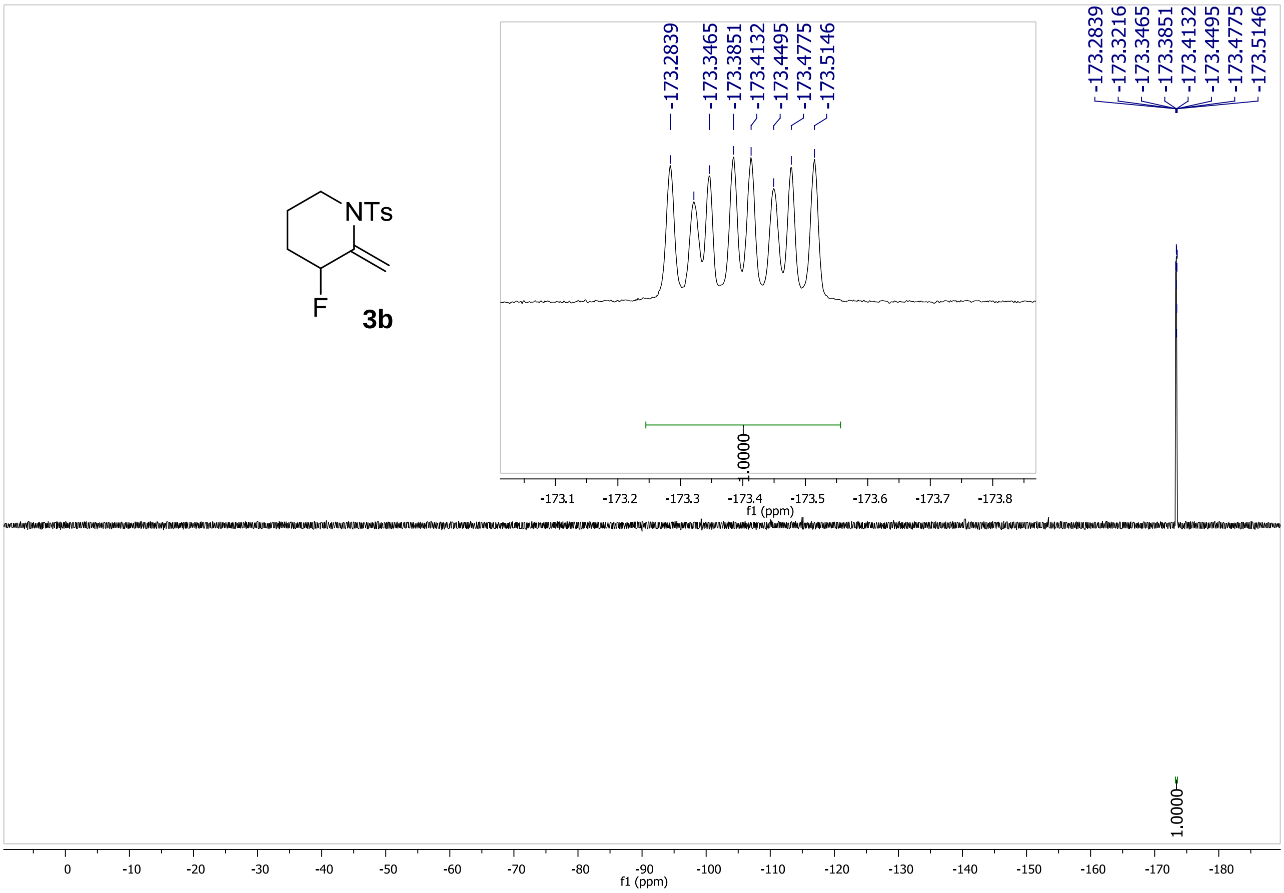
NTs
F
3b
-173.2839
-173.3465
-173.3851
-173.4132
-173.4495
-173.4775
-173.5146
1.0000
f1 (ppm)
-173.2839
-173.3216
-173.3465
-173.3851
-173.4132
-173.4495
-173.4775
-173.5146
1.0000
f1 (ppm)

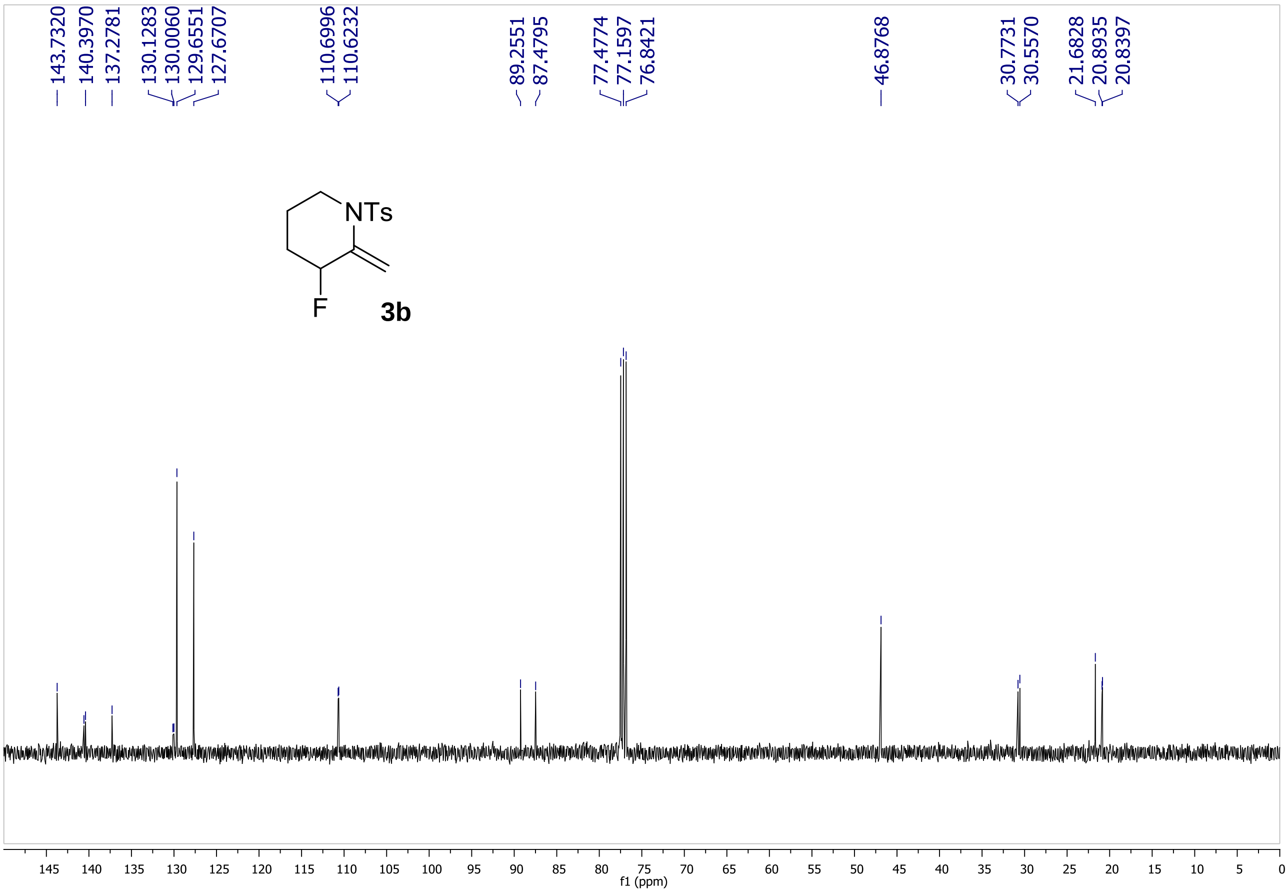
143.7320
140.3970
137.2781
130.1283
130.0060
129.6551
127.6707
110.6996
110.6232
89.2551
87.4795
77.4774
77.1597
76.8421
46.8768
30.7731
30.5570
21.6828
20.8935
20.8397
NTs
F
3b
f1 (ppm)

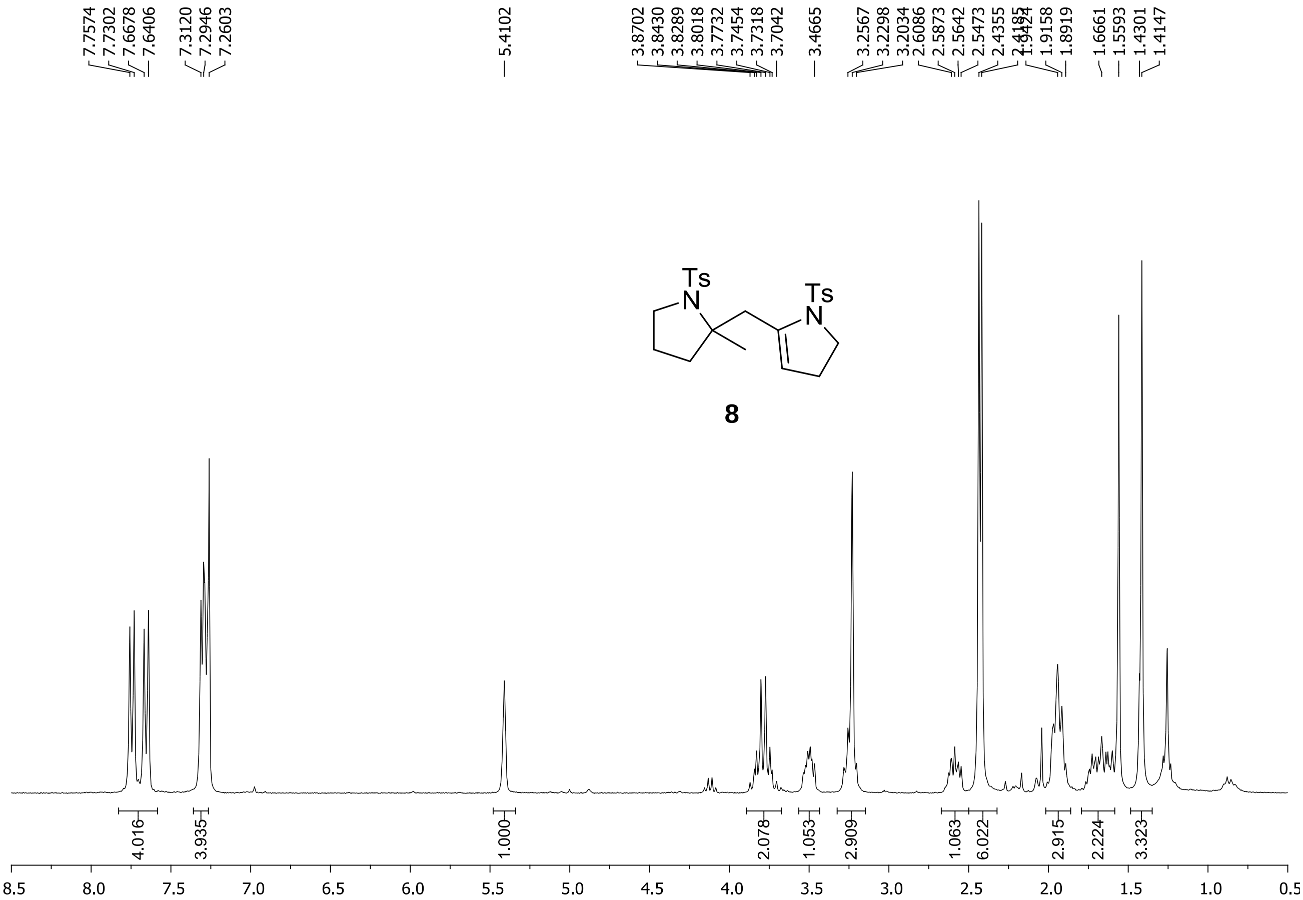
7.7574
7.7302
7.6678
7.6406
7.3120
7.2946
7.2603
5.4102
3.8702
3.8430
3.8289
3.8018
3.7732
3.7454
3.7318
3.7042
3.4665
3.2567
3.2298
3.2034
2.6086
2.5873
2.5642
2.5473
2.4355
1.9158
1.8919
1.6661
1.5593
1.4301
1.4147
Ts
N
Ts
N
8
4.016
3.935
1.000
2.078
1.053
2.909
1.063
6.022
2.915
2.224
3.323
8.5
8.0
7.5
7.0
6.5
6.0
5.5
5.0
4.5
4.0
3.5
3.0
2.5
2.0
1.5
1.0

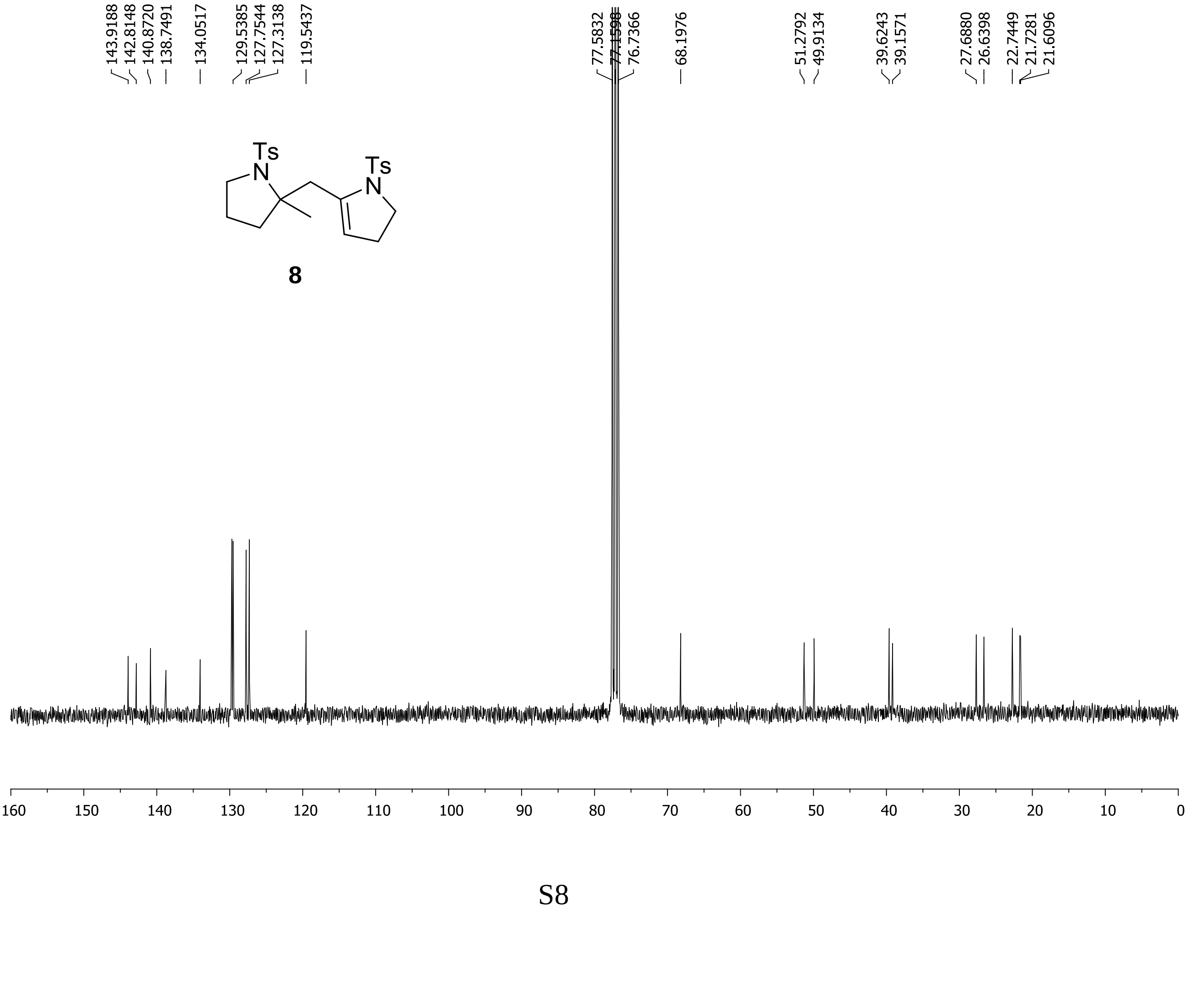
143.9188
142.8148
140.8720
138.7491
134.0517
129.5385
127.7544
127.3138
119.5437
77.5832
76.7366
68.1976
51.2792
49.9134
39.6243
39.1571
27.6880
26.6398
22.7449
21.7281
21.6096
Ts
N
Ts
N
8
160
150
140
130
120
110
100
90
80
70
60
50
40
30
20
10
0
